# Skeletal and Dentoalveolar Changes With Mandibular Expansion in Growing Children

**DOI:** 10.7759/cureus.47723

**Published:** 2023-10-26

**Authors:** Vaibhav Gandhi, Farheen Malek, Shivam Mehta, Aditya Tadinada, Robert Goldman, Sumit Yadav

**Affiliations:** 1 Orthodontics and Dentofacial Orthopaedics, University of Louisville, Louisville, USA; 2 Prosthodontics, Louisiana State University (LSU) School of Dentistry, New Orleans, USA; 3 Orthodontics, Marquette University School of Dentistry, Milwaukee, USA; 4 Oral and Maxillofacial Radiology, University of Connecticut, Farmington, USA; 5 Orthodontics, University of Connecticut, Farmington, USA; 6 Growth and Development, University of Nebraska Medical Center, Lincoln, USA

**Keywords:** cone-beam computed tomography, cone-beam computed tomography (cbct), mixed dentition, mandible, phase 1 orthodontic treatment, mandibular expansion

## Abstract

Introduction

The primary objective of this study was to quantitatively analyze the skeletal and dentoalveolar parameters following the mandibular expansion with a banded appliance. It was also part of the study to evaluate the amount of dental expansion and assess the change in the intermolar and individual first molar angulation. The basal bone parameters were compared to assess the skeletal effect of removable mandibular expansion appliance therapy.

Methods

In this retrospective cone beam computed tomography (CBCT) study, a total of 80 subjects with mandibular expansion therapy were screened. After imposing inclusion/exclusion criteria, 70 patients (40 females and 30 males) with a mean age of 8.8±1.24 years and 4.79±3.59 months were included. The mean expansion period was 3.04±1.61 months. Skeletal parameters such as buccal cortical thickness, buccal bone width, and cortical density were measured at 2mm from the alveolar crest, mid-root, and apex region in the coronal slice at the level of the mesiobuccal root of the first molar. Expansion parameters such as intermolar width, intermolar angulation, and individual molar angulation were also measured in the same slice. Finally, basal bone parameters such as inter-mental foramina distance and anterior arch perimeter were recorded.

Results

No significant difference (p>0.05) was found for most skeletal parameters following the expansion, except for the mid-root buccal bone width (p<0.05). On average, 4.54±2.53 mm of dental expansion (p<0.05) was achieved at the first molar region. Individual molar angulation showed a statistically significant difference (right = 7.46±7.91°, left = 7.53±7.18°, p=<0.05). The basal bone parameters showed no significant difference (p>0.05).

Conclusions

The mandibular expansion device leads to an increase in intermolar distance. The amount of expansion achieved with such devices is due to the buccal tipping of the molars. Skeletal effects such as cortical thickness, buccal bone width, or changes in the basal bone dimensions should not be expected with mandibular expansion therapy.

## Introduction

Dental crowding due to arch length-tooth size discrepancy is the most prevalent type of malocclusion, especially in the mandibular arch [1-2]. During the early mixed-dentition phase, when the permanent mandibular lateral incisors replace the primary incisors, on average, 1.6 mm of additional space is needed for the ideal alignment of the four mandibular incisors [3-5]. This is achieved either by an increase in the intercanine width, forward positioning of permanent incisors relative to the primary incisors, or slightly distal movement of primary canines towards the primate space [3,4]. However, if the discrepancy is more than 1.6 mm, these mechanisms might not be sufficient, and crowding is almost inevitable [6].

During the early mixed-dentition phase, the arch dimension has a significant influence on lower anterior crowding. Sayin and Turkkahraman observed larger deciduous intercanine and intermolar widths in patients without lower anterior crowding [6]. Mills found that patients with dental crowding had 4 mm narrower dental arches in the premolar region and reported a significant correlation between malalignment and arch width [7]. Sanin and Savara also observed wider mandibular arches in children who did not have crowding [8]. Hagberg associated the crowding with the intercanine distance of less than 26 mm in children between the ages of seven and 10 years, and there is no risk of crowding if the distance is more than 28 mm [9]. All this evidence suggests that during the early mixed-dentition period, expansion of the mandibular arch had some clinical implications for resolving possible mandibular crowding.

As compared to maxillary transverse deficiency, mandibular deficiency has little evidence in the literature [10]. Maxillary arch expansion is a common clinical practice to treat transverse maxillary deficiency; however, mandibular arch expansion is controversial and considered a less effective treatment regime. One factor could be the anatomical limitation since the maxilla has midpalatal sutures and the mandible does not [10]. Thus, any attempt to expand the mandibular arch is believed to be dentoalveolar tipping [11,12]. Maki et al. studied mandibular expansion using a Bihelix appliance and achieved considerable lateral expansion in children [12]. O’Grady et al. achieved more than 3 mm of mandibular expansion using the Schwarz appliance along with rapid maxillary expansion (RME) [13]. However, there are limited data available to provide insights about post-expansion changes in the molar angulation and the predictors for mandibular expansion. Additionally, information regarding the effect of mandibular arch expansion on the alveolar bone surrounding mandibular molars and on the basal bone is not known. Histological studies have shown that there may be a correlation between the density of bone and its anchorage potential, as well as the rate of tooth movement [14-16]. However, the information regarding the density of the cortical bone and how it affects mandibular arch expansion is missing from the current literature.

The primary objective of this study was to quantitatively analyze the cortical thickness, buccal bone width, and cortical density following the mandibular expansion with a banded appliance. It was also part of the study to evaluate the amount of dental expansion and assess the change in the intermolar and individual first molar angulation. Finally, the basal bone parameters were compared to evaluate the skeletal effect of removable mandibular expansion appliance therapy. Our null hypothesis was that there is no difference in the bone parameters (cortical thickness, bone width, cortical density), expansion parameters (amount of expansion, intermolar angulation, individual molar angulation), and basal bone parameters with mandibular arch expansion.

## Materials and methods

An institutional review board exemption was obtained for evaluating cone-beam computed tomography (CBCT) volumes retrieved from private practice. This retrospective study reviewed pretreatment and post-expansion CBCT scans of 80 patients who were referred for orthodontic treatment. All CBCT-scanned images were de-identified for protected health information (PHI) by authorized personnel from the Department of Oral and Maxillofacial Radiology at the University of Connecticut, Farmington, USA before being used as a study subject.

**Figure 1:**
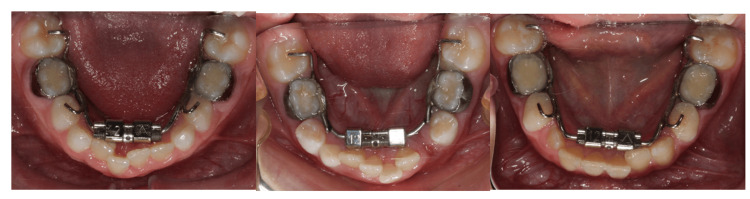
Images showing the design of the fixed mandibular expansion appliance used in this study

Patients were in growing status with age ranges from seven years to 12 years and presented with mandibular anterior crowding of greater than 3 mm (population). A fixed expander banded to the primary lower second molars was used for all the patients (Figure 1, intervention).

Occlusion rests were incorporated on the lower primary and permanent first molars to prevent overeruption. A quarter (1/4)-turn activation was conducted every alternate day until the required expansion was achieved. Angular and linear measurements were made on pretreatment and post-expansion CBCT scans for comparison (comparison). The amount of dentoalveolar expansion was measured on the mandibular permanent first molar (angular and linear), and the amount of skeletal expansion was measured on the mandibular basal bone (outcome). The detailed method for the measurement is explained below.

**Figure 2:**
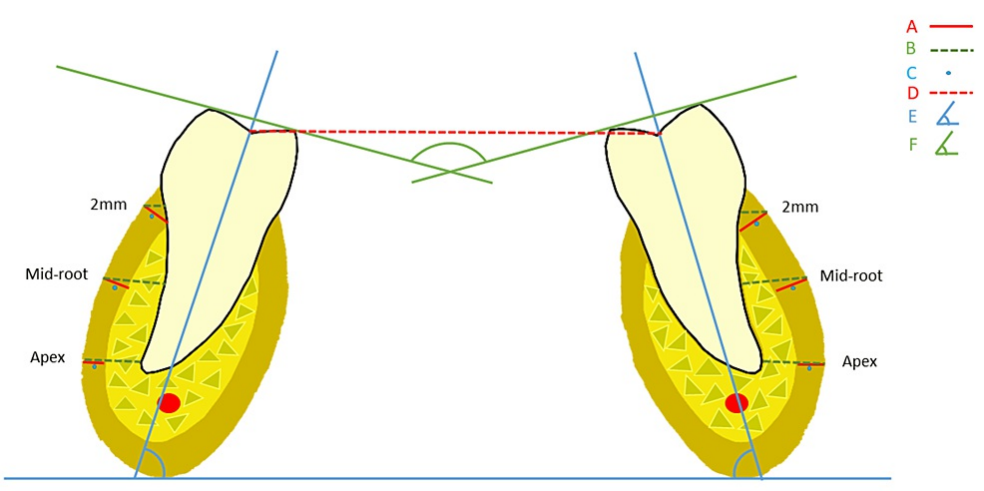
Diagram showing the method of measuring various dental and skeletal parameters used in this study. A: cortical bone thickness, B: buccal bone width, C: cortical density, D: intermolar width; E: individual molar angulation; F: intermolar angulation. This image was created by one of the authors to explain the study methodology.

Patients' age, gender, and dates for the beginning and end of the expansion were recorded. Exclusion criteria were missing records, poor quality of CBCT images, a higher amount of metallic noise from the metallic appliances, and an incomplete field of view. The CBCT scans were acquired using the i-CAT Next Generation (Imaging Sciences International, Hatfield, PA) CBCT unit. A standardized protocol of the iCAT for the extended (17 × 23 cm) field of view (FOV) with 0.3-mm slice thickness and 26.9-s acquisition time was used. All scans were saved in the DICOM-3 format and imported into Invivo5 ver. 5.3 (Anatomage Inc., San Jose, CA) software to be evaluated by a single examiner independently. The investigator reviewed the images on a split-screen dual-display monitor (HP Compaq LA2205wg, HP, Inc., Palo Alto, CA) under standardized conditions of ambient light and sound. The investigator had the full capability to evaluate the volumes in all three orthogonal planes and manipulate contrast and histogram (Figure 2).

Once the scans were imported into the reconstruction program, they were aligned using the three following steps (Figure 2): Step 1: First of all, scans were aligned in the field of view on axial sections at the level of the mandibular permanent first molar furcation area; Step 2: As our primary objective was to evaluate the mandibular permanent first molar region, we considered the mesial root as a reference to make measurements. Thus, all measurements were taken after aligning the sagittal section to get the mesial root in an upright position; Step 3: Finally, all the measurements were made in a coronal section bilaterally. Once the center was established on all three planes (axial, sagittal, and coronal) using the toggled crosshairs in the program, alveolar bone measurements were made in a coronal plane at the level of the medial root of the first permanent molar bilaterally, as described above.

**Figure 3:**
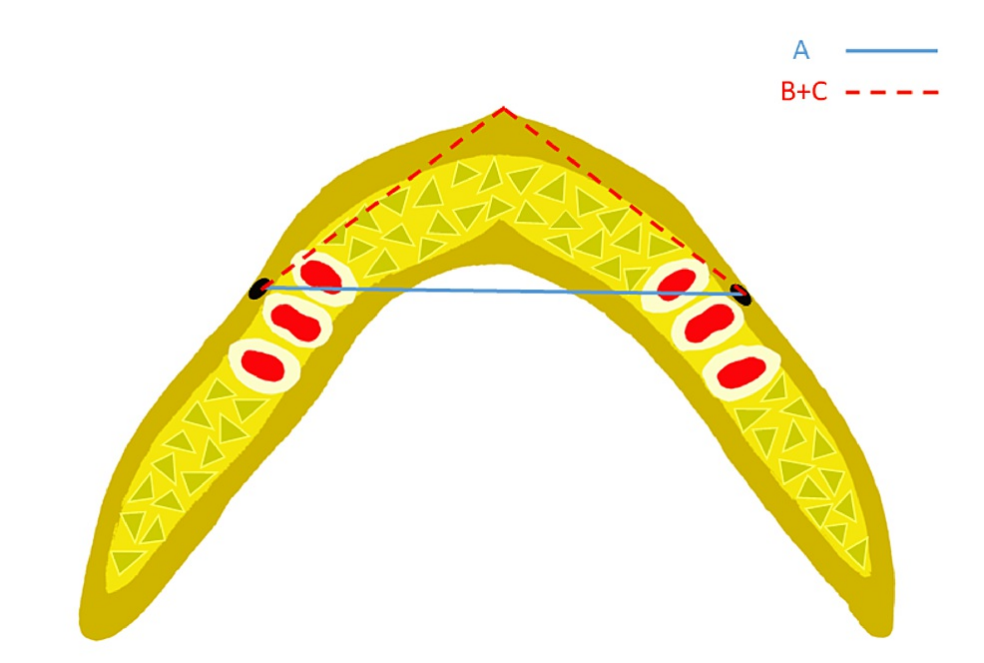
Diagram showing the method used for measuring the intermental foramina distance (A) and anterior arch perimeter (B+C). This image was created by one of the authors to explain the study methodology.

For alveolar bone measurements, buccal cortical bone thickness, as well as buccal bone width, were measured at 2 mm from the alveolar crest, mid-root, and at the level of the apex. Dense outer cortical bone was considered cortical bone thickness, and the distance between the root and outer surface was regarded as buccal bone width. Additionally, on the coronal view, cortical bone density was evaluated by measuring the pixel intensity value (PIV) equivalent to the Hounsfield unit scale bilaterally at the same locations as mentioned above (Figure 3).

For the dental parameters’ molar width (dental expansion), individual molar angulation and intermolar angulation were recorded. The distance between the central fossae of the right and left first molars was defined as molar width, and the differences between pretreatment and post-expansion molar widths were recorded as the amount of dental expansion. The angle between the long axis of the molar and the line passing through the lower border of the mandible was recorded bilaterally for the individual molar angulation. The angle between the right and left planes connecting the mesiobuccal and mesiolingual cusps of mandibular first molars were regarded as intermolar angulation.

To evaluate the basal bone parameters, bilateral mental foramina was considered a reference. The axial slice was moved until the mental foramina was clearly visible. In cases where only one mental foramen was visible, the coronal slice was adjusted until bilateral mental foramina were visible in the frame. The distance between the right and left mental foramen was considered basal bone width. The anterior arch perimeter was recorded as the total distance from the one mental foramen to the anterior-most point and from that point to the other mental foramen.

Statistical analysis

Simple descriptive statistics were used to summarize the data. Mean, standard deviation, and percentile distributions were computed for alveolar parameters (cortical thickness and alveolar bone width and density at 2 mm, mid-root, and apex), dental parameters (intermolar distance, intermolar angulation, and molar angulation), and basal bone parameters (basal bone width and anterior arch perimeter). For all the outcomes, intra- and inter-examiner reliability was computed by Krippendorff’s alpha. A one-sample Kolmogorov-Smirnov test was used to examine the normality of the distribution of bone width and bone height at different locations. All the measurements were normally distributed. A paired sample t-test was used for the pretreatment and post-expansion comparisons. Multivariate regression model analysis was used to investigate the factors associated with the amount of expansion achieved. All statistical tests were two-sided, and a P-value of <.05 was deemed to be statistically significant. Statistical analyses were computed using Prism software (GraphPad Software, La Jolla, CA).

## Results

After imposing selection criteria, a total of 70 patients (40 females and 30 males) were included in the study. The average age of the patient was 8.8±1.24 years and 4.79±3.59 months, and the mean expansion period was 3.04±1.61 months. Krippendorff’s alpha for intra-rater reliability was 0.94 and 0.91 for linear and density measurements, whereas for inter-rater reliability, it was 0.89 and 0.8 for linear and density measurements, respectively.

Alveolar bone parameters

**Figure 4:**
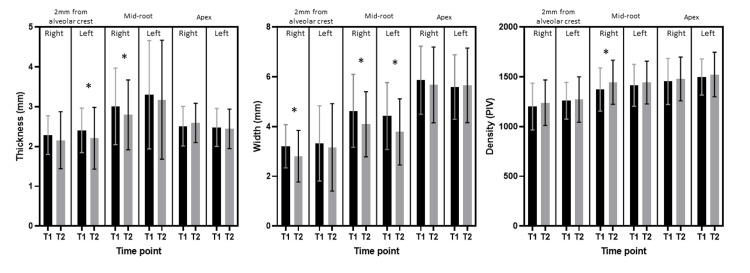
Intergroup comparisons of pretreatment and post-expansion cortical bone thickness, buccal bone width, and cortical density at 2 mm from the alveolar crest, mid-root, and apex bilaterally (paired sample t-test, *statistically significant at p<0.05)

The distribution of cortical thickness, bone width, and cortical density at 2 mm from the alveolar crest, mid-root, and apex is summarized in Table 1 for pretreatment and Table 2 for the post-expansion period, and comparisons are reported in Table 3 and Figure 4.

**Table 1:** Descriptive statistics about pretreatment cortical thickness, bone width, cortical density, and expansion parameters SD: standard deviation; SE: standard error; CI: confidence interval; PIV: pixel intensity value

Cortical thickness (mm)									
Location		Mean	SD	SE	95% CI (Lower)	95% CI (Upper)	Minimum	Maximum	Range
2 mm from alveolar crest	Right	2.30	0.49	0.06	2.20	2.40	1.30	3.70	2.40
	Left	2.40	0.56	0.07	2.30	2.50	1.30	3.80	2.50
Mid-root	Right	3.00	0.96	0.12	2.80	3.20	1.80	7.30	5.60
	Left	3.30	1.40	0.16	3.00	3.60	1.70	7.60	6.00
Apex	Right	2.50	0.50	0.06	2.40	2.60	1.30	4.30	3.00
	Left	2.50	0.48	0.06	2.40	2.60	1.70	4.20	2.50
Bone width (mm)									
Location		Mean	SD	SE	95% CI (Lower)	95% CI (Upper)	Minimum	Maximum	Range
2 mm from alveolar crest	Right	3.20	0.87	0.10	3.00	3.40	2.00	6.20	4.20
	Left	3.30	1.50	0.18	3.00	3.70	1.60	7.30	5.70
Mid-root	Right	4.60	1.50	0.18	4.30	5.00	2.20	8.50	6.30
	Left	4.40	1.30	0.16	4.10	4.70	2.00	6.90	4.90
Apex	Right	5.90	1.40	0.16	5.50	6.20	2.70	9.40	6.70
	Left	5.60	1.30	0.16	5.30	5.90	2.60	8.60	6.00
Cortical density (PIV)									
Location		Mean	SD	SE	95% CI (Lower)	95% CI (Upper)	Minimum	Maximum	Range
2 mm from alveolar crest	Right	1202	235	28	1145	1258	612	1746	1134
	Left	1260	185	22	1216	1304	786	1731	945
Mid-root	Right	1373	217	26	1320	1425	833	1851	1018
	Left	1415	210	25	1364	1465	950	1977	1027
Apex	Right	1454	231	28	1399	1510	745	2029	1284
	Left	1498	181	22	1455	1542	1104	1957	853
Expansion parameter									
Location		Mean	SD	SE	95% CI (Lower)	95% CI (Upper)	Minimum	Maximum	Range
Intermolar width (mm)		40	2.3	0.28	39	40	35	45	10
Intermolar angulation (degree)		159	12	1.5	156	162	126	180	54
Molar angulation (degree)	Right	75	6.9	0.82	73	76	56	87	31
	Left	74	7.6	0.91	72	76	54	92	38
Intermental foramina distance (mm)		40	2.2	0.26	40	41	35	45	11
Anterior arch perimeter (mm)		48	4.3	0.51	47	49	25	58	33

**Table 2:** Descriptive statistics about post-expansion cortical thickness, bone width, cortical density, and expansion parameters SD: standard deviation; SE: standard error; CI: confidence interval; PIV: pixel intensity value

Cortical thickness (mm)									
Location		Mean	SD	SE	95% CI (Lower)	95% CI (Upper)	Minimum	Maximum	Range
2 mm from alveolar crest	Right	2.20	0.72	0.09	2.00	2.30	0.03	5.10	5.00
	Left	2.20	0.78	0.09	2.00	2.40	0.03	3.90	3.90
Mid-root	Right	2.80	0.88	0.10	2.60	3.00	1.40	5.50	4.20
	Left	3.20	1.50	0.18	2.80	3.50	1.20	7.10	6.00
Apex	Right	2.60	0.49	0.06	2.50	2.70	1.20	4.10	2.90
	Left	2.40	0.50	0.06	2.30	2.60	1.60	3.90	2.40
Bone width (mm)									
Location		Mean	SD	SE	95% CI (Lower)	95% CI (Upper)	Minimum	Maximum	Range
2 mm from alveolar crest	Right	2.80	1.00	0.12	2.60	3.10	0.15	5.40	5.20
	Left	3.20	1.80	0.21	2.70	3.60	0.23	7.10	6.90
Mid-root	Right	4.10	1.30	0.16	3.80	4.40	1.80	6.60	4.80
	Left	3.80	1.30	0.16	3.50	4.10	1.40	7.30	6.00
Apex	Right	5.70	1.50	0.18	5.30	6.00	2.70	9.30	6.70
	Left	5.70	1.50	0.18	5.30	6.00	2.50	9.50	7.00
Cortical density (PIV)									
Location		Mean	SD	SE	95% CI (Lower)	95% CI (Upper)	Minimum	Maximum	Range
2 mm from alveolar crest	Right	1240	228	27	1185	1295	700	1686	986
	Left	1272	229	28	1217	1327	593	1681	1088
Mid-root	Right	1446	222	27	1392	1499	864	1854	990
	Left	1443	215	26	1391	1494	839	1853	1014
Apex	Right	1479	220	27	1426	1532	869	2055	1186
	Left	1524	224	27	1471	1578	883	1972	1089
Expansion parameter									
Location		Mean	SD	SE	95% CI (Lower)	95% CI (Upper)	Minimum	Maximum	Range
Intermolar width (mm)		44.0	3.1	0.4	43.0	45.0	37.0	51.0	14.0
Intermolar angulation (degree)		169.0	10.0	1.2	166.0	171.0	142.0	202.0	60.0
Molar angulation (degree)	Right	82.0	7.5	0.9	80.0	84.0	52.0	109.0	57.0
	Left	81.0	7.2	0.9	80.0	83.0	63.0	101.0	39.0
Intermental foramina distance (mm)		41.0	2.2	0.3	40.0	41.0	35.0	46.0	11.0
Anterior arch perimeter (mm)		49.0	3.0	0.4	48.0	50.0	41.0	56.0	15.0

**Table 3:** Comparison of pretreatment and post-expansion cortical thickness, bone width, cortical density, and expansion parameters SD: standard deviation; CI: confidence interval; PIV: pixel intensity value

Cortical thickness (mm)						
Location		Mean difference	SD	95% CI difference	R-squared	P-value
2 mm from alveolar crest	Right	-0.13	0.67	-0.2891 to 0.03054	0.036	0.1112
	Left	-0.20	0.64	-0.3511 to -0.04575	0.089	0.0116
Mid-root	Right	-0.21	0.80	-0.4048 to -0.02203	0.067	0.0294
	Left	-0.13	0.91	-0.3423 to 0.09205	0.019	0.2543
Apex	Right	0.08	0.44	-0.02181 to 0.1884	0.035	0.1185
	Left	-0.04	0.52	-0.1602 to 0.08903	0.005	0.5708
Bone width (mm)						
Location		Mean difference	SD	95% CI difference	R squared	P-value
2 mm from alveolar crest	Right	-0.40	0.90	-0.6121 to -0.1831	0.165	0.0004
	Left	-0.16	0.97	-0.3890 to 0.07444	0.026	0.1801
Mid-root	Right	-0.54	1.37	-0.8653 to -0.2144	0.137	0.0015
	Left	-0.64	1.07	-0.8913 to -0.3827	0.266	<0.0001
Apex	Right	-0.19	1.40	-0.5235 to 0.1447	0.018	0.2619
	Left	0.07	1.06	-0.1826 to 0.3215	0.004	0.5844
Bone density (PIV)						
Location		Mean difference	SD	95% CI difference	R squared	P-value
2 mm from alveolar crest	Right	38.52	283.4	-29.56 to 106.6	0.018	0.2628
	Left	12.42	256.2	-49.13 to 73.97	0.002	0.6885
Mid-root	Right	73.04	285.3	4.515 to 141.6	0.062	0.0371
	Left	28.06	277.4	-38.58 to 94.70	0.010	0.4037
Apex	Right	24.87	266.8	-39.22 to 88.96	0.009	0.4414
	Left	26.23	290.5	-43.56 to 96.02	0.008	0.4558
Expansion parameters						
Location		Mean difference	SD	95% CI difference	R squared	P-value
Intermolar width (mm)		4.54	2.53	3.935 to 5.144	0.765	<0.0001
Intermolar angulation (degree)		9.74	11.06	7.106 to 12.38	0.441	<0.0001
Molar angulation (degree)	Right	7.46	7.91	5.578 to 9.348	0.475	<0.0001
	Left	7.53	7.18	5.822 to 9.244	0.528	<0.0001
Intermental foramina distance (mm)		0.20	1.17	-0.07473 to 0.4810	0.030	0.1493
Anterior arch perimeter (mm)		0.63	3.74	-0.2615 to 1.522	0.028	0.1631

The buccal cortical thickness was found to be reduced at the post-expansion time point, but for the majority of locations, the difference was not statistically significant (p>0.05). Similarly, the buccal bone width was reduced post-expansion, and the difference was statistically significant at the mid-root level bilaterally (mean difference: right = -0.55±1.37 mm; left = -0.64±1.07 mm; p<0.001). The cortical density increased for all the locations, but the difference was not statistically significant (p>0.05).

Expansion parameters

Descriptive statistics about the pretreatment and post-expansion dentoalveolar parameters (intermolar width, intermolar angulation, and right and left molar angulation) and basal bone parameters (intermental foramina distance and anterior arch parameters) are reported in Tables 1 and 2. Pretreatment intermolar width was 40±2.3 mm, and intermolar angulation was 159±12°; post-expansion intermolar width was 44±3.1 mm, and intermolar angulation was 169±10° (Tables 1 and 2). Comparison of intermolar width and angulation showed a statistically significant difference (width: mean difference = 4.54±2.53 mm; angulation = 9.74±11.06°, p-value = <0.0001) (Table 3). Therefore, the expansion at the mandibular permanent first molar of 4.54±2.53 mm was achieved.

**Figure 5:**
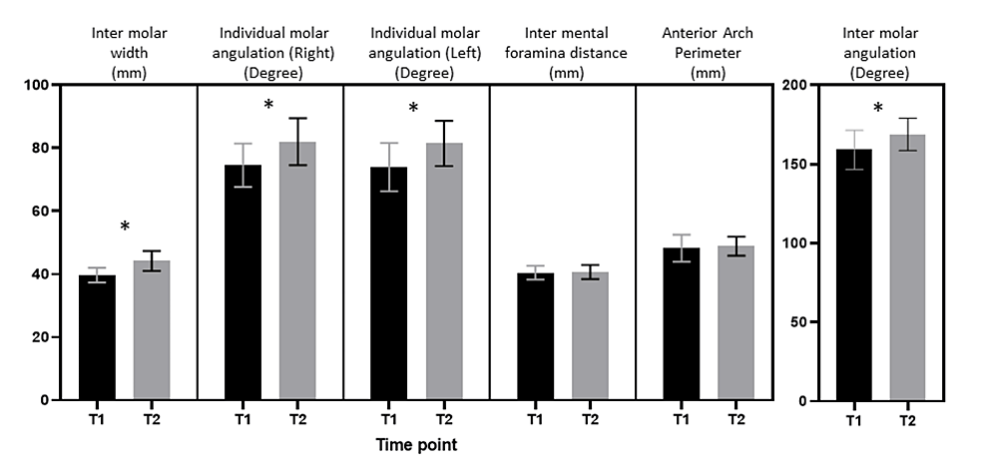
Intergroup comparisons of pretreatment and post-expansion intermolar width, individual molar angulation (right and left), intermental foramina distance, anterior arch perimeter, and intermolar angulation (paired sample t-test, *statistically significant at p<0.05)

The comparison of pretreatment and post-expansion molar angulation showed a statistically significant difference (mean difference: right = 7.46±7.91°, left = 7.53±7.18°, p-value = <0.0001) (Tables 1 and 2). On the comparison of the basal bone parameters (intermental foramina distance and anterior arch perimeters), no significant difference was found between pretreatment and post-expansion measurements (mean difference: intermental foramina distance = 0.2±1.17 mm, p-value = 0.1493; anterior arch perimeter = 0.63±3.74 mm, p-value = 0.1631) (Tables 1-3, Figure 5).

Based on the multivariate regression analysis, none of the parameters showed a significant value as a successful predictor for the amount of expansion achieved (p-value >0.05) (Table 4).

**Table 4:** A multiple linear regression model evaluates pretreatment predictability factors for the expansion achieved.

Variable	Estimate	Standard error	95% Confidence interval	P-value
Intercept	10.13	9.267	-8.461 to 28.71	0.2795
Intermolar width	-0.3481	0.2234	-0.7963 to 0.1000	0.1252
Intermolar angulation	-0.04629	0.04094	-0.1284 to 0.03582	0.2633
Molar angulation right	0.04859	0.07873	-0.1093 to 0.2065	0.5398
Molar angulation left	0.1087	0.08498	-0.06169 to 0.2792	0.2062
Intermolar foramen distance	0.1797	0.272	-0.3659 to 0.7254	0.5117
Anterior perimeter	-0.07098	0.1075	-0.2865 to 0.1446	0.5118
Cortical thickness (2 mm)	0.5721	1.177	-1.789 to 2.933	0.629
Cortical thickness (mid-root)	0.1493	0.571	-0.9959 to 1.295	0.7947
Cortical thickness (apex)	-0.6134	0.8076	-2.233 to 1.007	0.4509
Bone width (2 mm)	-0.09733	0.5283	-1.157 to 0.9623	0.8545
Bone width (mid-root)	0.03761	0.3219	-0.6081 to 0.6834	0.9075
Bone width (apex)	-0.06859	0.3796	-0.8300 to 0.6928	0.8573
Density (2 mm)	0.001137	0.002113	-0.003102 to 0.005376	0.5929
Density (mid-root)	-0.00183	0.002107	-0.006056 to 0.002396	0.389
Density (apex)	0.0009418	0.002084	-0.003239 to 0.005123	0.6532

## Discussion

The current retrospective study assessed the dentoalveolar and skeletal effects of mandibular expansion therapy using CBCT images. The null hypothesis was rejected as we found a significant difference between the cortical thickness, buccal bone width, intermolar width, intermolar angulation, and individual molar angulation in comparison of pretreatment with post-expansion. Most of the significant findings were associated with the dentoalveolar parameters and changes in molar angulation, with little or no effect on the basal bone.

The early treatment concept was developed based on the philosophy of correcting existing or developing dentoalveolar, skeletal, or soft tissue imbalances and establishing a favorable oral environment for the eruption of permanent dentition. Initiating phase I orthodontic or orthopedic therapy at a younger age may lead to phase II therapy, with simple orthodontic corrections for future abnormalities in the occlusion, thus avoiding the potential need for complex orthodontic treatment, including extraction or orthognathic surgery [17]. 

In this study, patients in a range of seven to 12 years with a mean age of 8.8±1.24 months were selected. Patients in this age range were chosen because the widening of both jaws, including dentoalveolar segments, should be completed before the adolescent growth spurt, preferably. A decrease in the intercanine width is expected after the age of 12 [18]. Also, the literature showed that the maxillary arch grows wider compared to the mandibular arch [17,19,20]. For these reasons, it is considered more apt to expand the arch width at a younger age to adapt dental, skeletal, and musculature so permanent teeth can erupt in their normal position [21]. If an appliance is used by actively growing children, a favorable response can be expected.

The commonly assessed parameter for mandibular arch expansion studies is intermolar width [22]. In the present study, we achieved 4.54±2.53 mm of expansion in the first molar region, measured from the central fossa of the first molars. This result is further supported by Quinzi et al., who reported 5.0±1.8 and 5.3±1.6 mm of expansion using Schwartz appliances with two different activation protocols [23]. Tai et al. observed a mean increase in intermolar width of 5.41 mm at the crown level, 4.39 mm at the cemento-enamel junction (CEJ), and 2.40 mm at the root level using a removable Schwartz expander [17,24]. O’Grady et al. observed 3.1 mm of expansion with the removable appliance [13]. This significant but comparatively smaller expansion could be due to patient compliance, differences in appliance activation, or differences in the method of assessment. However, the current study and evidence suggest that mandibular expansion increases the intermolar width, at least in terms of the dentoalveolar segment.

The effect of the mandibular arch expansion is limited to the dentoalveolar segment, usually resulting from the molar tipping [11,12]. The current study quantitatively evaluated such an effect by measuring the intermolar angulation and individual molar angulations of the first molars. We observed 9.74±11.06° changes in the intermolar angulation and 7.46±7.91° and 7.53±7.18° changes in the individual molar angulation for the right and left sides, respectively. There was no significant difference between the right and left molar angulations. Motoyoshi et al. reported an increase in the mean mandibular first molar inclination by 10.16±3.83° [25]. Tai et al. observed 8.7±1.4° tipping of the mandibular first molar using the Schwartz appliance [24]. In comparison with previous studies, our data showed no significant differences.

Buccal bone thickness can be an important parameter while undertaking expansion. It has been shown that the thickness of buccal bone for maxillary incisors should be greater than 2 mm, and decreased thickness of buccal alveolar bone may predispose to fenestration and recession [26]. However, not enough evidence is available for mandibular molars. The results of our study indicate that the buccal bone width for mandibular molars after the fixed expander in our study was found to be from 2.8 to 5.7 at different locations. Thus, even though molar tipping was evident after mandibular expansion, the buccal bone width was not significantly decreased by 2 mm and the apex. However, in the mid-root region, the buccal bone width decreased significantly by 0.54±1.37 mm on the left and 0.64±1.07 mm on the right side.

Literature suggests that the mandibular expansion devices have little skeletal effect [10-13, 17]. We considered mental foramina as a reference for this study as they are easy to identify. Since the study duration was only 3.04±1.61 months, we did not expect any significant change in their location solely due to the growth. In the current study, we found a non-significant difference of 0.20±1.17 mm in the intermental foramina distance and 0.63±3.4 mm in the anterior arch perimeter. This finding is supported by Milad et al., who reported a 0.54 to 0.46 mm increase in the mandibular body width following the expansion therapy, and this change was not statistically significant [10]. Furthermore, except for the bone at the mid-root level, all other parameters were statistically non-significant in the current study, strengthening the belief that a minimum skeletal effect is expected following the mandibular arch expansion. Also, multivariate regression analysis reported that none of the parameters (dental or skeletal) could be used to predict the amount of expansion.

Because no scientific research is perfect, this study has its own limitations. Due to its retrospective nature, all the measurements were made on previously acquired CBCT images. A prospective study with randomly allocated expansion and control groups of similar age and sex would be considered a more robust methodology. Furthermore, the amount of crowding before and after expansion and the post-treatment evaluation of the expansion parameters will provide a detailed explanation of the purpose and long-term stability of the expansion. Besides these limitations, this article has provided a comprehensive assessment of the dental and skeletal effects of the fixed expansion device, with the largest sample size so far. It will give readers a better understanding of the impact of expansion on bilateral molar angulation, bone width, and skeletal changes.

## Conclusions

The mandibular expansion therapy in the early mixed dentition phase using a banded appliance leads to a significant increase in the intermolar distance. No significant effect on the buccal bone quality (cortical thickness, buccal bone width, or cortical density) can be expected with the mandibular arch expansion. The amount of expansion achieved with such devices is purely dentoalveolar in nature and results from the equal amount of buccal tipping of the right and left molars. The effect of the expansion on the mandibular basal bone is minimal or non-significant.
